# Supplementary material for: Perceived impact of information signals on opinions about gluten-free diets
Source: PLoS One. 2021 Apr 8;16(4):e0248570. doi: 10.1371/journal.pone.0248570 (PMC8031409; doi:10.1371/journal.pone.0248570)
Supplement: S1 Appendix — (DOCX) [file pone.0248570.s001.docx]

Identity Theory 2 and Gluten Web of Belief, Qualtrics

Survey Flow

EmbeddedData

opp = Qual2911-1022USAGenPop

ridValue will be set from Panel or URL.

RISNValue will be set from Panel or URL.

Branch: New Branch

If

If Quota Overall 1550 Has Been Met

EmbeddedData

gc = 3

term = OQoverall

EndSurvey: Advanced

Block: Intro (2 Questions)

EmbeddedData

psid = ${e://Field/psid}

pid = ${e://Field/pid}

Branch: New Branch

If

If Are you 18 or older and wish to take the survey? I am younger than 18 or do not wish to take the survey. Is Selected

EmbeddedData

gc = 2

term = consent

EndSurvey: Advanced

Standard: demos (14 Questions)

Branch: New Branch

If

If What is your age? Less than 18 Is Selected

EmbeddedData

gc = 2

term = age

EndSurvey: Advanced

Branch: New Branch

If

If 50 States, D.C. and Puerto Rico I do not reside in the United States Is Selected

EmbeddedData

gc = 2

term = US

EndSurvey: Advanced

Standard: intro2 (1 Question)

BlockRandomizer: 1 -

Group: A

Block: I2 (2 Questions)

Block: R2 (2 Questions)

Group: B

Block: R2 (2 Questions)

Block: I2 (2 Questions)

Group: E

Block: I6 (6 Questions)

Block: R6 (6 Questions)

Group: F

Block: R6 (6 Questions)

Block: I6 (6 Questions)

Group: vegans

BlockRandomizer: 1 - Evenly Present Elements

Group: vegans R first

BlockRandomizer: 1 - Evenly Present Elements

Block: vegans R3 (3 Questions)

Block: vegans Rs alt (3 Questions)

Block: vegans I3 (3 Questions)

Group: vegan I first

Block: vegans I3 (3 Questions)

BlockRandomizer: 1 - Evenly Present Elements

Block: vegans R3 (3 Questions)

Block: vegans Rs alt (3 Questions)

Block: web ques (4 Questions)

Standard: web intro (1 Question)

BlockRandomizer: 10 - Evenly Present Elements

Standard: wactivist (11 Questions)

Standard: wceleb (11 Questions)

Standard: wday (11 Questions)

Standard: wdoctor (11 Questions)

Standard: wfriends (11 Questions)

Standard: wnews (11 Questions)

Standard: wper (11 Questions)

Standard: wsocial (11 Questions)

Standard: wmedia (11 Questions)

Standard: wstore (11 Questions)

Branch: New Branch

If

If Quota Overall 1550 Has Been Met

EmbeddedData

gc = 3

term = OQoverall

EndSurvey: Advanced

Branch: New Branch

If

If Q_TotalDuration Is Less Than or Equal to 200

EmbeddedData

gc = 4

term = speeder

EndSurvey: Advanced

EmbeddedData

gc = 1

LS = $e{(${e://Field/RISN}%3402)*3}

EndSurvey: Advanced

| Page Break |  |
| --- | --- |

Start of Block: Intro

Q101 This is a survey conducted by the Department of Agricultural Economics at Oklahoma State University. Its purpose is to measure people's attitudes towards a variety of social issues and food products. Your answers to the questions are anonymous, and at no point will we ask for contact information. The survey takes only about ten minutes to complete.


The principal investigator is Dr. Norwood, and for more information about the study he can be reached at bailey.norwood@okstate.edu. For more information on your rights as a survey respondent you may contact the OSU's Institutional Review Board at irb@okstate.edu or 405-744-3377.


You must be 18 years of age or older to take this survey. If you are over 18 and wish to take the survey, please click below and proceed to answer all questions.

Q102 Are you 18 or older and wish to take the survey?

- I am 18 years of age or older and wish to take the survey. (1)
- I am younger than 18 or do not wish to take the survey. (2)

Skip To: End of Block If Are you 18 or older and wish to take the survey? = I am younger than 18 or do not wish to take the survey.

End of Block: Intro

Start of Block: demos

Q104 Please tell us a few things about yourself, and remember your answers are confidential.

Q106 What is your age?

- Less than 18 (1)
- 18-24 (2)
- 25-34 (3)
- 35-44 (4)
- 45-54 (5)
- 55-64 (6)
- 65 or older (7)

Skip To: End of Block If What is your age? = Less than 18

Q108 What is your gender?

- Female (1)
- Male (2)
- Other (3)

| 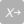 |
| --- |

Q110 What is your marital status?

- Married (1)
- Divorced (2)
- Widowed (3)
- I have a life partner but am not married (4)
- Separated but still married (5)
- Never married (6)
- Other (7)

Q112 What is your annual (pre-tax) household income (income from all earners who reside at your house)?

- less than $5,000 (1)
- $5,000 to $7,499 (2)
- $7,500 to $9,999 (3)
- $10,000 to $12,499 (4)
- $12,500 to $14,999 (5)
- $15,000 to $19,999 (6)
- $20,000 to $24,999 (7)
- $25,000 to $29,999 (8)
- $30,000 to $34,999 (9)
- $35,000 to $39,999 (10)
- $40,000 to $49,999 (11)
- $50,000 to $59,999 (12)
- $60,000 to $74,999 (13)
- $75,000 to $99,999 (14)
- $100,000 to $149,999 (15)
- $150,000 or more (16)

Q114 In which state do you currently reside?

▼ Alabama (1) ... I do not reside in the United States (53)

Skip To: End of Block If 50 States, D.C. and Puerto Rico = I do not reside in the United States

Q116 How many people reside in your household (including yourself and all ages)?

- 1 (I live alone) (1)
- 2 (2)
- 3 (3)
- 4 (4)
- 5 (5)
- 6 (6)
- more than 6 (7)

| 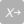 |
| --- |

Q118 How many people under the age of 18 reside in your household?

- 0 (1)
- 1 (8)
- 2 (2)
- 3 (3)
- 4 (4)
- 5 (5)
- 6 (6)
- more than 6 (7)

Q120 Is anyone in your household unemployed but looking for work?

- Yes (1)
- No (2)

Q122 Is anyone in your household fully employed?

- Yes (1)
- No (2)

Q124 Does your household rent or own your place of residence?

- Rent (1)
- Owned or being bought by a household member (2)
- Other (please specify) (3) ________________________________________________

Q130 Are you Hispanic?

- Yes (1)
- No (2)

| 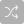 |
| --- |

Q128
Which of the following best describes your race or ethnicity?
Please check ALL that apply to you.

- American Indian or Alaska Native (1)
- Asian (2)
- Black or African American (3)
- Native Hawaiian (5)
- Other Pacific Islander (6)
- White (7)
- Other (8)

Q132 What is your highest level of education?

- No high school diploma (1)
- High school diploma (2)
- Associate's degree (3)
- Bachelor's degree (4)
- Graduate degree (5)

End of Block: demos

Start of Block: intro2

Q133 The first part of this survey consists of statements  where you are asked the degree to which you agree or disagree with each statement. Please answer each question honestly and to the best of your ability.

End of Block: intro2

Start of Block: I2

Q300 Citizens have a moral obligation to vote.

- Strongly disagree (1)
- Disagree (2)
- Somewhat disagree (3)
- Neither agree nor disagree (4)
- Somewhat agree (5)
- Agree (6)
- Strongly agree (7)

Q301 Not voting is an insult to those who died protecting democracy.

- Strongly disagree (1)
- Disagree (2)
- Somewhat disagree (3)
- Neither agree nor disagree (4)
- Somewhat agree (5)
- Agree (6)
- Strongly agree (7)

End of Block: I2

Start of Block: R2

Q302 I always vote.

- Strongly disagree (1)
- Disagree (2)
- Somewhat disagree (3)
- Neither agree nor disagree (4)
- Somewhat agree (5)
- Agree (6)
- Strongly agree (7)

Q303 I always research the political candidates before I vote.

- Strongly disagree (1)
- Disagree (2)
- Somewhat disagree (3)
- Neither agree nor disagree (4)
- Somewhat agree (5)
- Agree (6)
- Strongly agree (7)

End of Block: R2

Start of Block: I6

Q306 Citizens have a moral obligation to vote.

- Strongly disagree (1)
- Disagree (2)
- Somewhat disagree (3)
- Neither agree nor disagree (4)
- Somewhat agree (5)
- Agree (6)
- Strongly agree (7)

Q307 Not voting is an insult to those who died protecting democracy.

- Strongly disagree (1)
- Disagree (2)
- Somewhat disagree (3)
- Neither agree nor disagree (4)
- Somewhat agree (5)
- Agree (6)
- Strongly agree (7)

Q308 A good person should never gossip.

- Strongly disagree (1)
- Disagree (2)
- Somewhat disagree (3)
- Neither agree nor disagree (4)
- Somewhat agree (5)
- Agree (6)
- Strongly agree (7)

Q309 A good person should avoid listening to gossip.

- Strongly disagree (1)
- Disagree (2)
- Somewhat disagree (3)
- Neither agree nor disagree (4)
- Somewhat agree (5)
- Agree (6)
- Strongly agree (7)

Q310 I always try to be honest.

- Strongly disagree (1)
- Disagree (2)
- Somewhat disagree (3)
- Neither agree nor disagree (4)
- Somewhat agree (5)
- Agree (6)
- Strongly agree (7)

Q311 A person who lies cannot be trusted.

- Strongly disagree (1)
- Disagree (2)
- Somewhat disagree (3)
- Neither agree nor disagree (4)
- Somewhat agree (5)
- Agree (6)
- Strongly agree (7)

End of Block: I6

Start of Block: R6

Q312 I always vote.

- Strongly disagree (1)
- Disagree (2)
- Somewhat disagree (3)
- Neither agree nor disagree (4)
- Somewhat agree (5)
- Agree (6)
- Strongly agree (7)

Q313 I always research the political candidates before I vote.

- Strongly disagree (1)
- Disagree (2)
- Somewhat disagree (3)
- Neither agree nor disagree (4)
- Somewhat agree (5)
- Agree (6)
- Strongly agree (7)

Q314 I never gossip.

- Strongly disagree (1)
- Disagree (2)
- Somewhat disagree (3)
- Neither agree nor disagree (4)
- Somewhat agree (5)
- Agree (6)
- Strongly agree (7)

Q315 I never listen to gossip.

- Strongly disagree (1)
- Disagree (2)
- Somewhat disagree (3)
- Neither agree nor disagree (4)
- Somewhat agree (5)
- Agree (6)
- Strongly agree (7)

Q316 My friends would say I always tell the truth.

- Strongly disagree (1)
- Disagree (2)
- Somewhat disagree (3)
- Neither agree nor disagree (4)
- Somewhat agree (5)
- Agree (6)
- Strongly agree (7)

Q317 In the last month I have not told a lie.

- Strongly disagree (1)
- Disagree (2)
- Somewhat disagree (3)
- Neither agree nor disagree (4)
- Somewhat agree (5)
- Agree (6)
- Strongly agree (7)

End of Block: R6

Start of Block: vegans R3

Q324 I support a ban on the factory farming of animals.

- Strongly disagree (1)
- Disagree (2)
- Somewhat disagree (3)
- Neither agree nor disagree (4)
- Somewhat agree (5)
- Agree (6)
- Strongly agree (7)

Q325 I support a ban on slaughterhouses.

- Strongly disagree (1)
- Disagree (2)
- Somewhat disagree (3)
- Neither agree nor disagree (4)
- Somewhat agree (5)
- Agree (6)
- Strongly agree (7)

Q326 I support a ban on animal farming.

- Strongly disagree (1)
- Disagree (2)
- Somewhat disagree (3)
- Neither agree nor disagree (4)
- Somewhat agree (5)
- Agree (6)
- Strongly agree (7)

End of Block: vegans R3

Start of Block: vegans Rs alt

Q327 I support a ban on the factory farming of animals and will pay higher prices for food.

- Strongly disagree (1)
- Disagree (2)
- Somewhat disagree (3)
- Neither agree nor disagree (4)
- Somewhat agree (5)
- Agree (6)
- Strongly agree (7)

Q328 I support a ban on slaughterhouses and will stop eating meat.

- Strongly disagree (1)
- Disagree (2)
- Somewhat disagree (3)
- Neither agree nor disagree (4)
- Somewhat agree (5)
- Agree (6)
- Strongly agree (7)

Q329 I support a ban on animal farming and will stop eating meat, dairy, and eggs.

- Strongly disagree (1)
- Disagree (2)
- Somewhat disagree (3)
- Neither agree nor disagree (4)
- Somewhat agree (5)
- Agree (6)
- Strongly agree (7)

End of Block: vegans Rs alt

Start of Block: vegans I3

Q333 People should consume fewer animal-based foods (meat, dairy, and/or eggs) and more plant-based foods (fruits, grains, beans, and/or vegetables).

- Strongly disagree (1)
- Disagree (2)
- Somewhat disagree (3)
- Neither agree nor disagree (4)
- Somewhat agree (5)
- Agree (6)
- Strongly agree (7)

Q334 I have some discomfort with the way animals are used in the food industry.

- Strongly disagree (1)
- Disagree (2)
- Somewhat disagree (3)
- Neither agree nor disagree (4)
- Somewhat agree (5)
- Agree (6)
- Strongly agree (7)

Q335 Farmed animals have roughly the same ability to feel pain and discomfort as humans.

- Strongly disagree (1)
- Disagree (2)
- Somewhat disagree (3)
- Neither agree nor disagree (4)
- Somewhat agree (5)
- Agree (6)
- Strongly agree (7)

End of Block: vegans I3

Start of Block: web ques

Q260 The second section of this survey concerns foods containing gluten.

Q263 Have you ever heard of gluten-free food products?

- Yes (1)
- Maybe (2)
- No (3)

| 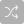 |
| --- |

Q261 Which of the following are most likely to contain gluten?

- Bread (1)
- Meat (2)
- Honey (3)
- Tomatoes (5)
- Lettuce (6)

| 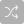 |
| --- |

Q262 What is your opinion about gluten?

- It is unhealthy for everyone (1)
- It is unhealthy for some people (2)
- It is healthy for everyone (3)
- Not sure (5)

End of Block: web ques

Start of Block: web intro

Q146 Next are ten questions. Each question will present you with a picture of a person and an event where they are exposed to information regarding gluten-free diets. For each person and event, please indicate how large of an impact you think it will have on the person's opinion of a gluten-free diet.

End of Block: web intro

Start of Block: wactivist

activist1

|  | Small Impact | Moderate Impact | Large Impact |
| --- | --- | --- | --- |

|  | 0 | 10 | 20 | 30 | 40 | 50 | 60 | 70 | 80 | 90 | 100 |
| --- | --- | --- | --- | --- | --- | --- | --- | --- | --- | --- | --- |

| What will be the impact of this event on this person's opinion of gluten-free diets? () | 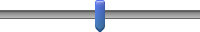 |
| --- | --- |

activist2

|  | Small Impact | Moderate Impact | Large Impact |
| --- | --- | --- | --- |

|  | 0 | 10 | 20 | 30 | 40 | 50 | 60 | 70 | 80 | 90 | 100 |
| --- | --- | --- | --- | --- | --- | --- | --- | --- | --- | --- | --- |

| What will be the impact of this event on this person's opinion of gluten-free diets? () | 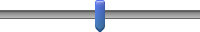 |
| --- | --- |

activist3

|  | Small Impact | Moderate Impact | Large Impact |
| --- | --- | --- | --- |

|  | 0 | 10 | 20 | 30 | 40 | 50 | 60 | 70 | 80 | 90 | 100 |
| --- | --- | --- | --- | --- | --- | --- | --- | --- | --- | --- | --- |

| What will be the impact of this event on this person's opinion of gluten-free diets? () | 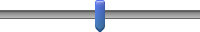 |
| --- | --- |

activist4

|  | Small Impact | Moderate Impact | Large Impact |
| --- | --- | --- | --- |

|  | 0 | 10 | 20 | 30 | 40 | 50 | 60 | 70 | 80 | 90 | 100 |
| --- | --- | --- | --- | --- | --- | --- | --- | --- | --- | --- | --- |

| What will be the impact of this event on this person's opinion of gluten-free diets? () | 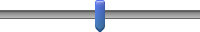 |
| --- | --- |

activist5

|  | Small Impact | Moderate Impact | Large Impact |
| --- | --- | --- | --- |

|  | 0 | 10 | 20 | 30 | 40 | 50 | 60 | 70 | 80 | 90 | 100 |
| --- | --- | --- | --- | --- | --- | --- | --- | --- | --- | --- | --- |

| What will be the impact of this event on this person's opinion of gluten-free diets? () | 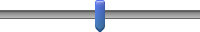 |
| --- | --- |

activist6

|  | Small Impact | Moderate Impact | Large Impact |
| --- | --- | --- | --- |

|  | 0 | 10 | 20 | 30 | 40 | 50 | 60 | 70 | 80 | 90 | 100 |
| --- | --- | --- | --- | --- | --- | --- | --- | --- | --- | --- | --- |

| What will be the impact of this event on this person's opinion of gluten-free diets? () | 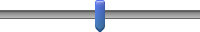 |
| --- | --- |

activist7

|  | Small Impact | Moderate Impact | Large Impact |
| --- | --- | --- | --- |

|  | 0 | 10 | 20 | 30 | 40 | 50 | 60 | 70 | 80 | 90 | 100 |
| --- | --- | --- | --- | --- | --- | --- | --- | --- | --- | --- | --- |

| What will be the impact of this event on this person's opinion of gluten-free diets? () | 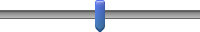 |
| --- | --- |

activist8

|  | Small Impact | Moderate Impact | Large Impact |
| --- | --- | --- | --- |

|  | 0 | 10 | 20 | 30 | 40 | 50 | 60 | 70 | 80 | 90 | 100 |
| --- | --- | --- | --- | --- | --- | --- | --- | --- | --- | --- | --- |

| What will be the impact of this event on this person's opinion of gluten-free diets? () | 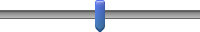 |
| --- | --- |

activist9

|  | Small Impact | Moderate Impact | Large Impact |
| --- | --- | --- | --- |

|  | 0 | 10 | 20 | 30 | 40 | 50 | 60 | 70 | 80 | 90 | 100 |
| --- | --- | --- | --- | --- | --- | --- | --- | --- | --- | --- | --- |

| What will be the impact of this event on this person's opinion of gluten-free diets? () | 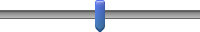 |
| --- | --- |

activist10

|  | Small Impact | Moderate Impact | Large Impact |
| --- | --- | --- | --- |

|  | 0 | 10 | 20 | 30 | 40 | 50 | 60 | 70 | 80 | 90 | 100 |
| --- | --- | --- | --- | --- | --- | --- | --- | --- | --- | --- | --- |

| What will be the impact of this event on this person's opinion of gluten-free diets? () | 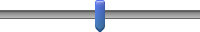 |
| --- | --- |

activist11

|  | Small Impact | Moderate Impact | Large Impact |
| --- | --- | --- | --- |

|  | 0 | 10 | 20 | 30 | 40 | 50 | 60 | 70 | 80 | 90 | 100 |
| --- | --- | --- | --- | --- | --- | --- | --- | --- | --- | --- | --- |

| What will be the impact of this event on this person's opinion of gluten-free diets? () | 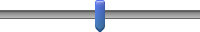 |
| --- | --- |

End of Block: wactivist

Start of Block: wceleb

celeb1

|  | Small Impact | Moderate Impact | Large Impact |
| --- | --- | --- | --- |

|  | 0 | 10 | 20 | 30 | 40 | 50 | 60 | 70 | 80 | 90 | 100 |
| --- | --- | --- | --- | --- | --- | --- | --- | --- | --- | --- | --- |

| What will be the impact of this event on this person's opinion of gluten-free diets? () | 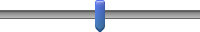 |
| --- | --- |

celeb2

|  | Small Impact | Moderate Impact | Large Impact |
| --- | --- | --- | --- |

|  | 0 | 10 | 20 | 30 | 40 | 50 | 60 | 70 | 80 | 90 | 100 |
| --- | --- | --- | --- | --- | --- | --- | --- | --- | --- | --- | --- |

| What will be the impact of this event on this person's opinion of gluten-free diets? () | 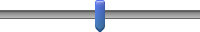 |
| --- | --- |

celeb3

|  | Small Impact | Moderate Impact | Large Impact |
| --- | --- | --- | --- |

|  | 0 | 10 | 20 | 30 | 40 | 50 | 60 | 70 | 80 | 90 | 100 |
| --- | --- | --- | --- | --- | --- | --- | --- | --- | --- | --- | --- |

| What will be the impact of this event on this person's opinion of gluten-free diets? () | 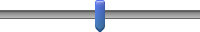 |
| --- | --- |

celeb4

|  | Small Impact | Moderate Impact | Large Impact |
| --- | --- | --- | --- |

|  | 0 | 10 | 20 | 30 | 40 | 50 | 60 | 70 | 80 | 90 | 100 |
| --- | --- | --- | --- | --- | --- | --- | --- | --- | --- | --- | --- |

| What will be the impact of this event on this person's opinion of gluten-free diets? () | 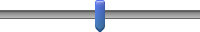 |
| --- | --- |

celeb5

|  | Small Impact | Moderate Impact | Large Impact |
| --- | --- | --- | --- |

|  | 0 | 10 | 20 | 30 | 40 | 50 | 60 | 70 | 80 | 90 | 100 |
| --- | --- | --- | --- | --- | --- | --- | --- | --- | --- | --- | --- |

| What will be the impact of this event on this person's opinion of gluten-free diets? () | 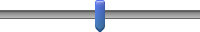 |
| --- | --- |

celeb6

|  | Small Impact | Moderate Impact | Large Impact |
| --- | --- | --- | --- |

|  | 0 | 10 | 20 | 30 | 40 | 50 | 60 | 70 | 80 | 90 | 100 |
| --- | --- | --- | --- | --- | --- | --- | --- | --- | --- | --- | --- |

| What will be the impact of this event on this person's opinion of gluten-free diets? () | 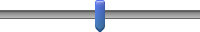 |
| --- | --- |

celeb7

|  | Small Impact | Moderate Impact | Large Impact |
| --- | --- | --- | --- |

|  | 0 | 10 | 20 | 30 | 40 | 50 | 60 | 70 | 80 | 90 | 100 |
| --- | --- | --- | --- | --- | --- | --- | --- | --- | --- | --- | --- |

| What will be the impact of this event on this person's opinion of gluten-free diets? () | 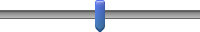 |
| --- | --- |

celeb8

|  | Small Impact | Moderate Impact | Large Impact |
| --- | --- | --- | --- |

|  | 0 | 10 | 20 | 30 | 40 | 50 | 60 | 70 | 80 | 90 | 100 |
| --- | --- | --- | --- | --- | --- | --- | --- | --- | --- | --- | --- |

| What will be the impact of this event on this person's opinion of gluten-free diets? () | 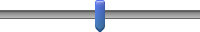 |
| --- | --- |

celeb9

|  | Small Impact | Moderate Impact | Large Impact |
| --- | --- | --- | --- |

|  | 0 | 10 | 20 | 30 | 40 | 50 | 60 | 70 | 80 | 90 | 100 |
| --- | --- | --- | --- | --- | --- | --- | --- | --- | --- | --- | --- |

| What will be the impact of this event on this person's opinion of gluten-free diets? () | 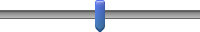 |
| --- | --- |

celeb10

|  | Small Impact | Moderate Impact | Large Impact |
| --- | --- | --- | --- |

|  | 0 | 10 | 20 | 30 | 40 | 50 | 60 | 70 | 80 | 90 | 100 |
| --- | --- | --- | --- | --- | --- | --- | --- | --- | --- | --- | --- |

| What will be the impact of this event on this person's opinion of gluten-free diets? () | 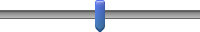 |
| --- | --- |

celeb11

|  | Small Impact | Moderate Impact | Large Impact |
| --- | --- | --- | --- |

|  | 0 | 10 | 20 | 30 | 40 | 50 | 60 | 70 | 80 | 90 | 100 |
| --- | --- | --- | --- | --- | --- | --- | --- | --- | --- | --- | --- |

| What will be the impact of this event on this person's opinion of gluten-free diets? () | 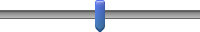 |
| --- | --- |

End of Block: wceleb

Start of Block: wday

day1

|  | Small Impact | Moderate Impact | Large Impact |
| --- | --- | --- | --- |

|  | 0 | 10 | 20 | 30 | 40 | 50 | 60 | 70 | 80 | 90 | 100 |
| --- | --- | --- | --- | --- | --- | --- | --- | --- | --- | --- | --- |

| What will be the impact of this event on this person's opinion of gluten-free diets? () | 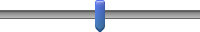 |
| --- | --- |

day2

|  | Small Impact | Moderate Impact | Large Impact |
| --- | --- | --- | --- |

|  | 0 | 10 | 20 | 30 | 40 | 50 | 60 | 70 | 80 | 90 | 100 |
| --- | --- | --- | --- | --- | --- | --- | --- | --- | --- | --- | --- |

| What will be the impact of this event on this person's opinion of gluten-free diets? () | 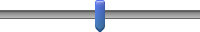 |
| --- | --- |

day3

|  | Small Impact | Moderate Impact | Large Impact |
| --- | --- | --- | --- |

|  | 0 | 10 | 20 | 30 | 40 | 50 | 60 | 70 | 80 | 90 | 100 |
| --- | --- | --- | --- | --- | --- | --- | --- | --- | --- | --- | --- |

| What will be the impact of this event on this person's opinion of gluten-free diets? () | 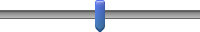 |
| --- | --- |

day4

|  | Small Impact | Moderate Impact | Large Impact |
| --- | --- | --- | --- |

|  | 0 | 10 | 20 | 30 | 40 | 50 | 60 | 70 | 80 | 90 | 100 |
| --- | --- | --- | --- | --- | --- | --- | --- | --- | --- | --- | --- |

| What will be the impact of this event on this person's opinion of gluten-free diets? () | 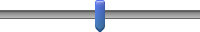 |
| --- | --- |

day5

|  | Small Impact | Moderate Impact | Large Impact |
| --- | --- | --- | --- |

|  | 0 | 10 | 20 | 30 | 40 | 50 | 60 | 70 | 80 | 90 | 100 |
| --- | --- | --- | --- | --- | --- | --- | --- | --- | --- | --- | --- |

| What will be the impact of this event on this person's opinion of gluten-free diets? () | 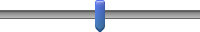 |
| --- | --- |

day6

|  | Small Impact | Moderate Impact | Large Impact |
| --- | --- | --- | --- |

|  | 0 | 10 | 20 | 30 | 40 | 50 | 60 | 70 | 80 | 90 | 100 |
| --- | --- | --- | --- | --- | --- | --- | --- | --- | --- | --- | --- |

| What will be the impact of this event on this person's opinion of gluten-free diets? () | 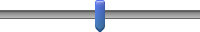 |
| --- | --- |

day7

|  | Small Impact | Moderate Impact | Large Impact |
| --- | --- | --- | --- |

|  | 0 | 10 | 20 | 30 | 40 | 50 | 60 | 70 | 80 | 90 | 100 |
| --- | --- | --- | --- | --- | --- | --- | --- | --- | --- | --- | --- |

| What will be the impact of this event on this person's opinion of gluten-free diets? () | 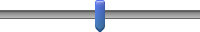 |
| --- | --- |

day8

|  | Small Impact | Moderate Impact | Large Impact |
| --- | --- | --- | --- |

|  | 0 | 10 | 20 | 30 | 40 | 50 | 60 | 70 | 80 | 90 | 100 |
| --- | --- | --- | --- | --- | --- | --- | --- | --- | --- | --- | --- |

| What will be the impact of this event on this person's opinion of gluten-free diets? () | 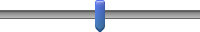 |
| --- | --- |

day9

|  | Small Impact | Moderate Impact | Large Impact |
| --- | --- | --- | --- |

|  | 0 | 10 | 20 | 30 | 40 | 50 | 60 | 70 | 80 | 90 | 100 |
| --- | --- | --- | --- | --- | --- | --- | --- | --- | --- | --- | --- |

| What will be the impact of this event on this person's opinion of gluten-free diets? () | 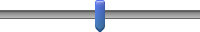 |
| --- | --- |

day10

|  | Small Impact | Moderate Impact | Large Impact |
| --- | --- | --- | --- |

|  | 0 | 10 | 20 | 30 | 40 | 50 | 60 | 70 | 80 | 90 | 100 |
| --- | --- | --- | --- | --- | --- | --- | --- | --- | --- | --- | --- |

| What will be the impact of this event on this person's opinion of gluten-free diets? () | 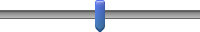 |
| --- | --- |

day11

|  | Small Impact | Moderate Impact | Large Impact |
| --- | --- | --- | --- |

|  | 0 | 10 | 20 | 30 | 40 | 50 | 60 | 70 | 80 | 90 | 100 |
| --- | --- | --- | --- | --- | --- | --- | --- | --- | --- | --- | --- |

| What will be the impact of this event on this person's opinion of gluten-free diets? () | 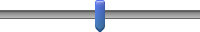 |
| --- | --- |

End of Block: wday

Start of Block: wdoctor

doctor1

|  | Small Impact | Moderate Impact | Large Impact |
| --- | --- | --- | --- |

|  | 0 | 10 | 20 | 30 | 40 | 50 | 60 | 70 | 80 | 90 | 100 |
| --- | --- | --- | --- | --- | --- | --- | --- | --- | --- | --- | --- |

| What will be the impact of this event on this person's opinion of gluten-free diets? () | 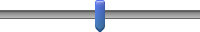 |
| --- | --- |

doctor2

|  | Small Impact | Moderate Impact | Large Impact |
| --- | --- | --- | --- |

|  | 0 | 10 | 20 | 30 | 40 | 50 | 60 | 70 | 80 | 90 | 100 |
| --- | --- | --- | --- | --- | --- | --- | --- | --- | --- | --- | --- |

| What will be the impact of this event on this person's opinion of gluten-free diets? () | 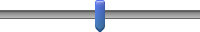 |
| --- | --- |

doctor3

|  | Small Impact | Moderate Impact | Large Impact |
| --- | --- | --- | --- |

|  | 0 | 10 | 20 | 30 | 40 | 50 | 60 | 70 | 80 | 90 | 100 |
| --- | --- | --- | --- | --- | --- | --- | --- | --- | --- | --- | --- |

| What will be the impact of this event on this person's opinion of gluten-free diets? () | 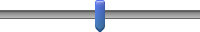 |
| --- | --- |

doctor4

|  | Small Impact | Moderate Impact | Large Impact |
| --- | --- | --- | --- |

|  | 0 | 10 | 20 | 30 | 40 | 50 | 60 | 70 | 80 | 90 | 100 |
| --- | --- | --- | --- | --- | --- | --- | --- | --- | --- | --- | --- |

| What will be the impact of this event on this person's opinion of gluten-free diets? () | 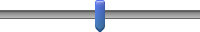 |
| --- | --- |

doctor5

|  | Small Impact | Moderate Impact | Large Impact |
| --- | --- | --- | --- |

|  | 0 | 10 | 20 | 30 | 40 | 50 | 60 | 70 | 80 | 90 | 100 |
| --- | --- | --- | --- | --- | --- | --- | --- | --- | --- | --- | --- |

| What will be the impact of this event on this person's opinion of gluten-free diets? () | 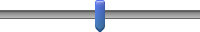 |
| --- | --- |

doctor6

|  | Small Impact | Moderate Impact | Large Impact |
| --- | --- | --- | --- |

|  | 0 | 10 | 20 | 30 | 40 | 50 | 60 | 70 | 80 | 90 | 100 |
| --- | --- | --- | --- | --- | --- | --- | --- | --- | --- | --- | --- |

| What will be the impact of this event on this person's opinion of gluten-free diets? () | 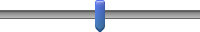 |
| --- | --- |

doctor7

|  | Small Impact | Moderate Impact | Large Impact |
| --- | --- | --- | --- |

|  | 0 | 10 | 20 | 30 | 40 | 50 | 60 | 70 | 80 | 90 | 100 |
| --- | --- | --- | --- | --- | --- | --- | --- | --- | --- | --- | --- |

| What will be the impact of this event on this person's opinion of gluten-free diets? () | 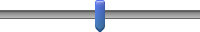 |
| --- | --- |

doctor8

|  | Small Impact | Moderate Impact | Large Impact |
| --- | --- | --- | --- |

|  | 0 | 10 | 20 | 30 | 40 | 50 | 60 | 70 | 80 | 90 | 100 |
| --- | --- | --- | --- | --- | --- | --- | --- | --- | --- | --- | --- |

| What will be the impact of this event on this person's opinion of gluten-free diets? () | 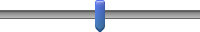 |
| --- | --- |

doctor9

|  | Small Impact | Moderate Impact | Large Impact |
| --- | --- | --- | --- |

|  | 0 | 10 | 20 | 30 | 40 | 50 | 60 | 70 | 80 | 90 | 100 |
| --- | --- | --- | --- | --- | --- | --- | --- | --- | --- | --- | --- |

| What will be the impact of this event on this person's opinion of gluten-free diets? () | 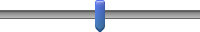 |
| --- | --- |

doctor10

|  | Small Impact | Moderate Impact | Large Impact |
| --- | --- | --- | --- |

|  | 0 | 10 | 20 | 30 | 40 | 50 | 60 | 70 | 80 | 90 | 100 |
| --- | --- | --- | --- | --- | --- | --- | --- | --- | --- | --- | --- |

| What will be the impact of this event on this person's opinion of gluten-free diets? () | 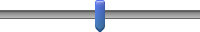 |
| --- | --- |

doctor11

|  | Small Impact | Moderate Impact | Large Impact |
| --- | --- | --- | --- |

|  | 0 | 10 | 20 | 30 | 40 | 50 | 60 | 70 | 80 | 90 | 100 |
| --- | --- | --- | --- | --- | --- | --- | --- | --- | --- | --- | --- |

| What will be the impact of this event on this person's opinion of gluten-free diets? () | 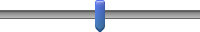 |
| --- | --- |

End of Block: wdoctor

Start of Block: wfriends

friends1

|  | Small Impact | Moderate Impact | Large Impact |
| --- | --- | --- | --- |

|  | 0 | 10 | 20 | 30 | 40 | 50 | 60 | 70 | 80 | 90 | 100 |
| --- | --- | --- | --- | --- | --- | --- | --- | --- | --- | --- | --- |

| What will be the impact of this event on this person's opinion of gluten-free diets? () | 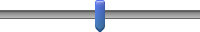 |
| --- | --- |

friends2

|  | Small Impact | Moderate Impact | Large Impact |
| --- | --- | --- | --- |

|  | 0 | 10 | 20 | 30 | 40 | 50 | 60 | 70 | 80 | 90 | 100 |
| --- | --- | --- | --- | --- | --- | --- | --- | --- | --- | --- | --- |

| What will be the impact of this event on this person's opinion of gluten-free diets? () | 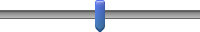 |
| --- | --- |

friends3

|  | Small Impact | Moderate Impact | Large Impact |
| --- | --- | --- | --- |

|  | 0 | 10 | 20 | 30 | 40 | 50 | 60 | 70 | 80 | 90 | 100 |
| --- | --- | --- | --- | --- | --- | --- | --- | --- | --- | --- | --- |

| What will be the impact of this event on this person's opinion of gluten-free diets? () | 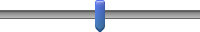 |
| --- | --- |

friends4

|  | Small Impact | Moderate Impact | Large Impact |
| --- | --- | --- | --- |

|  | 0 | 10 | 20 | 30 | 40 | 50 | 60 | 70 | 80 | 90 | 100 |
| --- | --- | --- | --- | --- | --- | --- | --- | --- | --- | --- | --- |

| What will be the impact of this event on this person's opinion of gluten-free diets? () | 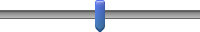 |
| --- | --- |

friends5

|  | Small Impact | Moderate Impact | Large Impact |
| --- | --- | --- | --- |

|  | 0 | 10 | 20 | 30 | 40 | 50 | 60 | 70 | 80 | 90 | 100 |
| --- | --- | --- | --- | --- | --- | --- | --- | --- | --- | --- | --- |

| What will be the impact of this event on this person's opinion of gluten-free diets? () | 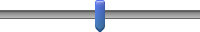 |
| --- | --- |

friends6

|  | Small Impact | Moderate Impact | Large Impact |
| --- | --- | --- | --- |

|  | 0 | 10 | 20 | 30 | 40 | 50 | 60 | 70 | 80 | 90 | 100 |
| --- | --- | --- | --- | --- | --- | --- | --- | --- | --- | --- | --- |

| What will be the impact of this event on this person's opinion of gluten-free diets? () | 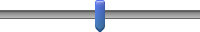 |
| --- | --- |

friends7

|  | Small Impact | Moderate Impact | Large Impact |
| --- | --- | --- | --- |

|  | 0 | 10 | 20 | 30 | 40 | 50 | 60 | 70 | 80 | 90 | 100 |
| --- | --- | --- | --- | --- | --- | --- | --- | --- | --- | --- | --- |

| What will be the impact of this event on this person's opinion of gluten-free diets? () | 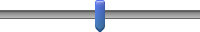 |
| --- | --- |

friends8

|  | Small Impact | Moderate Impact | Large Impact |
| --- | --- | --- | --- |

|  | 0 | 10 | 20 | 30 | 40 | 50 | 60 | 70 | 80 | 90 | 100 |
| --- | --- | --- | --- | --- | --- | --- | --- | --- | --- | --- | --- |

| What will be the impact of this event on this person's opinion of gluten-free diets? () | 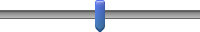 |
| --- | --- |

friends9

|  | Small Impact | Moderate Impact | Large Impact |
| --- | --- | --- | --- |

|  | 0 | 10 | 20 | 30 | 40 | 50 | 60 | 70 | 80 | 90 | 100 |
| --- | --- | --- | --- | --- | --- | --- | --- | --- | --- | --- | --- |

| What will be the impact of this event on this person's opinion of gluten-free diets? () | 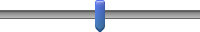 |
| --- | --- |

friends10

|  | Small Impact | Moderate Impact | Large Impact |
| --- | --- | --- | --- |

|  | 0 | 10 | 20 | 30 | 40 | 50 | 60 | 70 | 80 | 90 | 100 |
| --- | --- | --- | --- | --- | --- | --- | --- | --- | --- | --- | --- |

| What will be the impact of this event on this person's opinion of gluten-free diets? () | 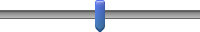 |
| --- | --- |

friends11

|  | Small Impact | Moderate Impact | Large Impact |
| --- | --- | --- | --- |

|  | 0 | 10 | 20 | 30 | 40 | 50 | 60 | 70 | 80 | 90 | 100 |
| --- | --- | --- | --- | --- | --- | --- | --- | --- | --- | --- | --- |

| What will be the impact of this event on this person's opinion of gluten-free diets? () | 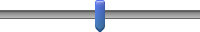 |
| --- | --- |

End of Block: wfriends

Start of Block: wnews

news1

|  | Small Impact | Moderate Impact | Large Impact |
| --- | --- | --- | --- |

|  | 0 | 10 | 20 | 30 | 40 | 50 | 60 | 70 | 80 | 90 | 100 |
| --- | --- | --- | --- | --- | --- | --- | --- | --- | --- | --- | --- |

| What will be the impact of this event on this person's opinion of gluten-free diets? () | 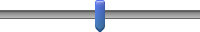 |
| --- | --- |

news2

|  | Small Impact | Moderate Impact | Large Impact |
| --- | --- | --- | --- |

|  | 0 | 10 | 20 | 30 | 40 | 50 | 60 | 70 | 80 | 90 | 100 |
| --- | --- | --- | --- | --- | --- | --- | --- | --- | --- | --- | --- |

| What will be the impact of this event on this person's opinion of gluten-free diets? () | 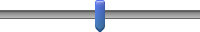 |
| --- | --- |

news3

|  | Small Impact | Moderate Impact | Large Impact |
| --- | --- | --- | --- |

|  | 0 | 10 | 20 | 30 | 40 | 50 | 60 | 70 | 80 | 90 | 100 |
| --- | --- | --- | --- | --- | --- | --- | --- | --- | --- | --- | --- |

| What will be the impact of this event on this person's opinion of gluten-free diets? () | 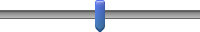 |
| --- | --- |

news4

|  | Small Impact | Moderate Impact | Large Impact |
| --- | --- | --- | --- |

|  | 0 | 10 | 20 | 30 | 40 | 50 | 60 | 70 | 80 | 90 | 100 |
| --- | --- | --- | --- | --- | --- | --- | --- | --- | --- | --- | --- |

| What will be the impact of this event on this person's opinion of gluten-free diets? () | 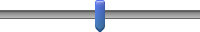 |
| --- | --- |

news5

|  | Small Impact | Moderate Impact | Large Impact |
| --- | --- | --- | --- |

|  | 0 | 10 | 20 | 30 | 40 | 50 | 60 | 70 | 80 | 90 | 100 |
| --- | --- | --- | --- | --- | --- | --- | --- | --- | --- | --- | --- |

| What will be the impact of this event on this person's opinion of gluten-free diets? () | 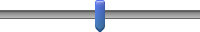 |
| --- | --- |

news6

|  | Small Impact | Moderate Impact | Large Impact |
| --- | --- | --- | --- |

|  | 0 | 10 | 20 | 30 | 40 | 50 | 60 | 70 | 80 | 90 | 100 |
| --- | --- | --- | --- | --- | --- | --- | --- | --- | --- | --- | --- |

| What will be the impact of this event on this person's opinion of gluten-free diets? () | 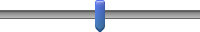 |
| --- | --- |

news7

|  | Small Impact | Moderate Impact | Large Impact |
| --- | --- | --- | --- |

|  | 0 | 10 | 20 | 30 | 40 | 50 | 60 | 70 | 80 | 90 | 100 |
| --- | --- | --- | --- | --- | --- | --- | --- | --- | --- | --- | --- |

| What will be the impact of this event on this person's opinion of gluten-free diets? () | 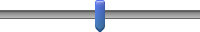 |
| --- | --- |

news8

|  | Small Impact | Moderate Impact | Large Impact |
| --- | --- | --- | --- |

|  | 0 | 10 | 20 | 30 | 40 | 50 | 60 | 70 | 80 | 90 | 100 |
| --- | --- | --- | --- | --- | --- | --- | --- | --- | --- | --- | --- |

| What will be the impact of this event on this person's opinion of gluten-free diets? () | 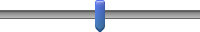 |
| --- | --- |

news9

|  | Small Impact | Moderate Impact | Large Impact |
| --- | --- | --- | --- |

|  | 0 | 10 | 20 | 30 | 40 | 50 | 60 | 70 | 80 | 90 | 100 |
| --- | --- | --- | --- | --- | --- | --- | --- | --- | --- | --- | --- |

| What will be the impact of this event on this person's opinion of gluten-free diets? () | 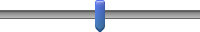 |
| --- | --- |

news10

|  | Small Impact | Moderate Impact | Large Impact |
| --- | --- | --- | --- |

|  | 0 | 10 | 20 | 30 | 40 | 50 | 60 | 70 | 80 | 90 | 100 |
| --- | --- | --- | --- | --- | --- | --- | --- | --- | --- | --- | --- |

| What will be the impact of this event on this person's opinion of gluten-free diets? () | 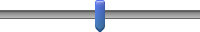 |
| --- | --- |

news11

|  | Small Impact | Moderate Impact | Large Impact |
| --- | --- | --- | --- |

|  | 0 | 10 | 20 | 30 | 40 | 50 | 60 | 70 | 80 | 90 | 100 |
| --- | --- | --- | --- | --- | --- | --- | --- | --- | --- | --- | --- |

| What will be the impact of this event on this person's opinion of gluten-free diets? () | 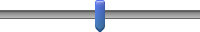 |
| --- | --- |

End of Block: wnews

Start of Block: wper

per1

|  | Small Impact | Moderate Impact | Large Impact |
| --- | --- | --- | --- |

|  | 0 | 10 | 20 | 30 | 40 | 50 | 60 | 70 | 80 | 90 | 100 |
| --- | --- | --- | --- | --- | --- | --- | --- | --- | --- | --- | --- |

| What will be the impact of this event on this person's opinion of gluten-free diets? () | 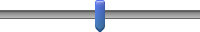 |
| --- | --- |

per2

|  | Small Impact | Moderate Impact | Large Impact |
| --- | --- | --- | --- |

|  | 0 | 10 | 20 | 30 | 40 | 50 | 60 | 70 | 80 | 90 | 100 |
| --- | --- | --- | --- | --- | --- | --- | --- | --- | --- | --- | --- |

| What will be the impact of this event on this person's opinion of gluten-free diets? () | 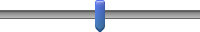 |
| --- | --- |

per3

|  | Small Impact | Moderate Impact | Large Impact |
| --- | --- | --- | --- |

|  | 0 | 10 | 20 | 30 | 40 | 50 | 60 | 70 | 80 | 90 | 100 |
| --- | --- | --- | --- | --- | --- | --- | --- | --- | --- | --- | --- |

| What will be the impact of this event on this person's opinion of gluten-free diets? () | 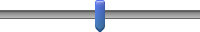 |
| --- | --- |

per4

|  | Small Impact | Moderate Impact | Large Impact |
| --- | --- | --- | --- |

|  | 0 | 10 | 20 | 30 | 40 | 50 | 60 | 70 | 80 | 90 | 100 |
| --- | --- | --- | --- | --- | --- | --- | --- | --- | --- | --- | --- |

| What will be the impact of this event on this person's opinion of gluten-free diets? () | 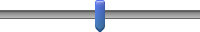 |
| --- | --- |

per5

|  | Small Impact | Moderate Impact | Large Impact |
| --- | --- | --- | --- |

|  | 0 | 10 | 20 | 30 | 40 | 50 | 60 | 70 | 80 | 90 | 100 |
| --- | --- | --- | --- | --- | --- | --- | --- | --- | --- | --- | --- |

| What will be the impact of this event on this person's opinion of gluten-free diets? () | 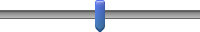 |
| --- | --- |

per6

|  | Small Impact | Moderate Impact | Large Impact |
| --- | --- | --- | --- |

|  | 0 | 10 | 20 | 30 | 40 | 50 | 60 | 70 | 80 | 90 | 100 |
| --- | --- | --- | --- | --- | --- | --- | --- | --- | --- | --- | --- |

| What will be the impact of this event on this person's opinion of gluten-free diets? () | 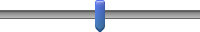 |
| --- | --- |

per7

|  | Small Impact | Moderate Impact | Large Impact |
| --- | --- | --- | --- |

|  | 0 | 10 | 20 | 30 | 40 | 50 | 60 | 70 | 80 | 90 | 100 |
| --- | --- | --- | --- | --- | --- | --- | --- | --- | --- | --- | --- |

| What will be the impact of this event on this person's opinion of gluten-free diets? () | 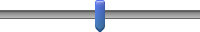 |
| --- | --- |

per8

|  | Small Impact | Moderate Impact | Large Impact |
| --- | --- | --- | --- |

|  | 0 | 10 | 20 | 30 | 40 | 50 | 60 | 70 | 80 | 90 | 100 |
| --- | --- | --- | --- | --- | --- | --- | --- | --- | --- | --- | --- |

| What will be the impact of this event on this person's opinion of gluten-free diets? () | 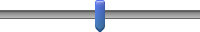 |
| --- | --- |

per9

|  | Small Impact | Moderate Impact | Large Impact |
| --- | --- | --- | --- |

|  | 0 | 10 | 20 | 30 | 40 | 50 | 60 | 70 | 80 | 90 | 100 |
| --- | --- | --- | --- | --- | --- | --- | --- | --- | --- | --- | --- |

| What will be the impact of this event on this person's opinion of gluten-free diets? () | 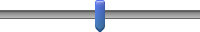 |
| --- | --- |

per10

|  | Small Impact | Moderate Impact | Large Impact |
| --- | --- | --- | --- |

|  | 0 | 10 | 20 | 30 | 40 | 50 | 60 | 70 | 80 | 90 | 100 |
| --- | --- | --- | --- | --- | --- | --- | --- | --- | --- | --- | --- |

| What will be the impact of this event on this person's opinion of gluten-free diets? () | 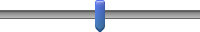 |
| --- | --- |

per11

|  | Small Impact | Moderate Impact | Large Impact |
| --- | --- | --- | --- |

|  | 0 | 10 | 20 | 30 | 40 | 50 | 60 | 70 | 80 | 90 | 100 |
| --- | --- | --- | --- | --- | --- | --- | --- | --- | --- | --- | --- |

| What will be the impact of this event on this person's opinion of gluten-free diets? () | 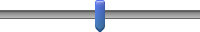 |
| --- | --- |

End of Block: wper

Start of Block: wsocial

social1

|  | Small Impact | Moderate Impact | Large Impact |
| --- | --- | --- | --- |

|  | 0 | 10 | 20 | 30 | 40 | 50 | 60 | 70 | 80 | 90 | 100 |
| --- | --- | --- | --- | --- | --- | --- | --- | --- | --- | --- | --- |

| What will be the impact of this event on this person's opinion of gluten-free diets? () | 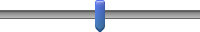 |
| --- | --- |

social2

|  | Small Impact | Moderate Impact | Large Impact |
| --- | --- | --- | --- |

|  | 0 | 10 | 20 | 30 | 40 | 50 | 60 | 70 | 80 | 90 | 100 |
| --- | --- | --- | --- | --- | --- | --- | --- | --- | --- | --- | --- |

| What will be the impact of this event on this person's opinion of gluten-free diets? () | 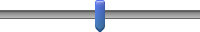 |
| --- | --- |

social3

|  | Small Impact | Moderate Impact | Large Impact |
| --- | --- | --- | --- |

|  | 0 | 10 | 20 | 30 | 40 | 50 | 60 | 70 | 80 | 90 | 100 |
| --- | --- | --- | --- | --- | --- | --- | --- | --- | --- | --- | --- |

| What will be the impact of this event on this person's opinion of gluten-free diets? () | 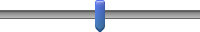 |
| --- | --- |

social4

|  | Small Impact | Moderate Impact | Large Impact |
| --- | --- | --- | --- |

|  | 0 | 10 | 20 | 30 | 40 | 50 | 60 | 70 | 80 | 90 | 100 |
| --- | --- | --- | --- | --- | --- | --- | --- | --- | --- | --- | --- |

| What will be the impact of this event on this person's opinion of gluten-free diets? () | 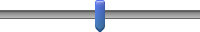 |
| --- | --- |

social5

|  | Small Impact | Moderate Impact | Large Impact |
| --- | --- | --- | --- |

|  | 0 | 10 | 20 | 30 | 40 | 50 | 60 | 70 | 80 | 90 | 100 |
| --- | --- | --- | --- | --- | --- | --- | --- | --- | --- | --- | --- |

| What will be the impact of this event on this person's opinion of gluten-free diets? () | 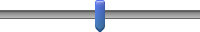 |
| --- | --- |

social6

|  | Small Impact | Moderate Impact | Large Impact |
| --- | --- | --- | --- |

|  | 0 | 10 | 20 | 30 | 40 | 50 | 60 | 70 | 80 | 90 | 100 |
| --- | --- | --- | --- | --- | --- | --- | --- | --- | --- | --- | --- |

| What will be the impact of this event on this person's opinion of gluten-free diets? () | 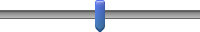 |
| --- | --- |

social7

|  | Small Impact | Moderate Impact | Large Impact |
| --- | --- | --- | --- |

|  | 0 | 10 | 20 | 30 | 40 | 50 | 60 | 70 | 80 | 90 | 100 |
| --- | --- | --- | --- | --- | --- | --- | --- | --- | --- | --- | --- |

| What will be the impact of this event on this person's opinion of gluten-free diets? () | 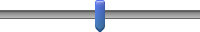 |
| --- | --- |

social8

|  | Small Impact | Moderate Impact | Large Impact |
| --- | --- | --- | --- |

|  | 0 | 10 | 20 | 30 | 40 | 50 | 60 | 70 | 80 | 90 | 100 |
| --- | --- | --- | --- | --- | --- | --- | --- | --- | --- | --- | --- |

| What will be the impact of this event on this person's opinion of gluten-free diets? () | 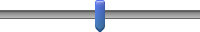 |
| --- | --- |

social9

|  | Small Impact | Moderate Impact | Large Impact |
| --- | --- | --- | --- |

|  | 0 | 10 | 20 | 30 | 40 | 50 | 60 | 70 | 80 | 90 | 100 |
| --- | --- | --- | --- | --- | --- | --- | --- | --- | --- | --- | --- |

| What will be the impact of this event on this person's opinion of gluten-free diets? () | 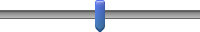 |
| --- | --- |

social10

|  | Small Impact | Moderate Impact | Large Impact |
| --- | --- | --- | --- |

|  | 0 | 10 | 20 | 30 | 40 | 50 | 60 | 70 | 80 | 90 | 100 |
| --- | --- | --- | --- | --- | --- | --- | --- | --- | --- | --- | --- |

| What will be the impact of this event on this person's opinion of gluten-free diets? () | 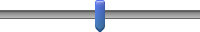 |
| --- | --- |

social11

|  | Small Impact | Moderate Impact | Large Impact |
| --- | --- | --- | --- |

|  | 0 | 10 | 20 | 30 | 40 | 50 | 60 | 70 | 80 | 90 | 100 |
| --- | --- | --- | --- | --- | --- | --- | --- | --- | --- | --- | --- |

| What will be the impact of this event on this person's opinion of gluten-free diets? () | 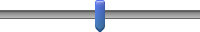 |
| --- | --- |

End of Block: wsocial

Start of Block: wmedia

media1

|  | Small Impact | Moderate Impact | Large Impact |
| --- | --- | --- | --- |

|  | 0 | 10 | 20 | 30 | 40 | 50 | 60 | 70 | 80 | 90 | 100 |
| --- | --- | --- | --- | --- | --- | --- | --- | --- | --- | --- | --- |

| What will be the impact of this event on this person's opinion of gluten-free diets? () | 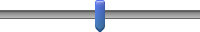 |
| --- | --- |

media2

|  | Small Impact | Moderate Impact | Large Impact |
| --- | --- | --- | --- |

|  | 0 | 10 | 20 | 30 | 40 | 50 | 60 | 70 | 80 | 90 | 100 |
| --- | --- | --- | --- | --- | --- | --- | --- | --- | --- | --- | --- |

| What will be the impact of this event on this person's opinion of gluten-free diets? () | 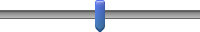 |
| --- | --- |

media3

|  | Small Impact | Moderate Impact | Large Impact |
| --- | --- | --- | --- |

|  | 0 | 10 | 20 | 30 | 40 | 50 | 60 | 70 | 80 | 90 | 100 |
| --- | --- | --- | --- | --- | --- | --- | --- | --- | --- | --- | --- |

| What will be the impact of this event on this person's opinion of gluten-free diets? () | 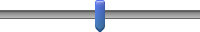 |
| --- | --- |

media4

|  | Small Impact | Moderate Impact | Large Impact |
| --- | --- | --- | --- |

|  | 0 | 10 | 20 | 30 | 40 | 50 | 60 | 70 | 80 | 90 | 100 |
| --- | --- | --- | --- | --- | --- | --- | --- | --- | --- | --- | --- |

| What will be the impact of this event on this person's opinion of gluten-free diets? () | 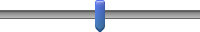 |
| --- | --- |

media5

|  | Small Impact | Moderate Impact | Large Impact |
| --- | --- | --- | --- |

|  | 0 | 10 | 20 | 30 | 40 | 50 | 60 | 70 | 80 | 90 | 100 |
| --- | --- | --- | --- | --- | --- | --- | --- | --- | --- | --- | --- |

| What will be the impact of this event on this person's opinion of gluten-free diets? () | 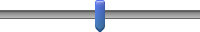 |
| --- | --- |

media6

|  | Small Impact | Moderate Impact | Large Impact |
| --- | --- | --- | --- |

|  | 0 | 10 | 20 | 30 | 40 | 50 | 60 | 70 | 80 | 90 | 100 |
| --- | --- | --- | --- | --- | --- | --- | --- | --- | --- | --- | --- |

| What will be the impact of this event on this person's opinion of gluten-free diets? () | 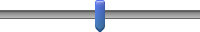 |
| --- | --- |

media7

|  | Small Impact | Moderate Impact | Large Impact |
| --- | --- | --- | --- |

|  | 0 | 10 | 20 | 30 | 40 | 50 | 60 | 70 | 80 | 90 | 100 |
| --- | --- | --- | --- | --- | --- | --- | --- | --- | --- | --- | --- |

| What will be the impact of this event on this person's opinion of gluten-free diets? () | 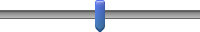 |
| --- | --- |

media8

|  | Small Impact | Moderate Impact | Large Impact |
| --- | --- | --- | --- |

|  | 0 | 10 | 20 | 30 | 40 | 50 | 60 | 70 | 80 | 90 | 100 |
| --- | --- | --- | --- | --- | --- | --- | --- | --- | --- | --- | --- |

| What will be the impact of this event on this person's opinion of gluten-free diets? () |  |
| --- | --- |

media9

|  | Small Impact | Moderate Impact | Large Impact |
| --- | --- | --- | --- |

|  | 0 | 10 | 20 | 30 | 40 | 50 | 60 | 70 | 80 | 90 | 100 |
| --- | --- | --- | --- | --- | --- | --- | --- | --- | --- | --- | --- |

| What will be the impact of this event on this person's opinion of gluten-free diets? () |  |
| --- | --- |

media10

|  | Small Impact | Moderate Impact | Large Impact |
| --- | --- | --- | --- |

|  | 0 | 10 | 20 | 30 | 40 | 50 | 60 | 70 | 80 | 90 | 100 |
| --- | --- | --- | --- | --- | --- | --- | --- | --- | --- | --- | --- |

| What will be the impact of this event on this person's opinion of gluten-free diets? () |  |
| --- | --- |

media11

|  | Small Impact | Moderate Impact | Large Impact |
| --- | --- | --- | --- |

|  | 0 | 10 | 20 | 30 | 40 | 50 | 60 | 70 | 80 | 90 | 100 |
| --- | --- | --- | --- | --- | --- | --- | --- | --- | --- | --- | --- |

| What will be the impact of this event on this person's opinion of gluten-free diets? () |  |
| --- | --- |

End of Block: wmedia

Start of Block: wstore

store1

|  | Small Impact | Moderate Impact | Large Impact |
| --- | --- | --- | --- |

|  | 0 | 10 | 20 | 30 | 40 | 50 | 60 | 70 | 80 | 90 | 100 |
| --- | --- | --- | --- | --- | --- | --- | --- | --- | --- | --- | --- |

| What will be the impact of this event on this person's opinion of gluten-free diets? () |  |
| --- | --- |

store2

|  | Small Impact | Moderate Impact | Large Impact |
| --- | --- | --- | --- |

|  | 0 | 10 | 20 | 30 | 40 | 50 | 60 | 70 | 80 | 90 | 100 |
| --- | --- | --- | --- | --- | --- | --- | --- | --- | --- | --- | --- |

| What will be the impact of this event on this person's opinion of gluten-free diets? () |  |
| --- | --- |

store3

|  | Small Impact | Moderate Impact | Large Impact |
| --- | --- | --- | --- |

|  | 0 | 10 | 20 | 30 | 40 | 50 | 60 | 70 | 80 | 90 | 100 |
| --- | --- | --- | --- | --- | --- | --- | --- | --- | --- | --- | --- |

| What will be the impact of this event on this person's opinion of gluten-free diets? () |  |
| --- | --- |

store4

|  | Small Impact | Moderate Impact | Large Impact |
| --- | --- | --- | --- |

|  | 0 | 10 | 20 | 30 | 40 | 50 | 60 | 70 | 80 | 90 | 100 |
| --- | --- | --- | --- | --- | --- | --- | --- | --- | --- | --- | --- |

| What will be the impact of this event on this person's opinion of gluten-free diets? () |  |
| --- | --- |

store5

|  | Small Impact | Moderate Impact | Large Impact |
| --- | --- | --- | --- |

|  | 0 | 10 | 20 | 30 | 40 | 50 | 60 | 70 | 80 | 90 | 100 |
| --- | --- | --- | --- | --- | --- | --- | --- | --- | --- | --- | --- |

| What will be the impact of this event on this person's opinion of gluten-free diets? () |  |
| --- | --- |

store6

|  | Small Impact | Moderate Impact | Large Impact |
| --- | --- | --- | --- |

|  | 0 | 10 | 20 | 30 | 40 | 50 | 60 | 70 | 80 | 90 | 100 |
| --- | --- | --- | --- | --- | --- | --- | --- | --- | --- | --- | --- |

| What will be the impact of this event on this person's opinion of gluten-free diets? () |  |
| --- | --- |

store7

|  | Small Impact | Moderate Impact | Large Impact |
| --- | --- | --- | --- |

|  | 0 | 10 | 20 | 30 | 40 | 50 | 60 | 70 | 80 | 90 | 100 |
| --- | --- | --- | --- | --- | --- | --- | --- | --- | --- | --- | --- |

| What will be the impact of this event on this person's opinion of gluten-free diets? () |  |
| --- | --- |

store8

|  | Small Impact | Moderate Impact | Large Impact |
| --- | --- | --- | --- |

|  | 0 | 10 | 20 | 30 | 40 | 50 | 60 | 70 | 80 | 90 | 100 |
| --- | --- | --- | --- | --- | --- | --- | --- | --- | --- | --- | --- |

| What will be the impact of this event on this person's opinion of gluten-free diets? () |  |
| --- | --- |

store9

|  | Small Impact | Moderate Impact | Large Impact |
| --- | --- | --- | --- |

|  | 0 | 10 | 20 | 30 | 40 | 50 | 60 | 70 | 80 | 90 | 100 |
| --- | --- | --- | --- | --- | --- | --- | --- | --- | --- | --- | --- |

| What will be the impact of this event on this person's opinion of gluten-free diets? () |  |
| --- | --- |

store10

|  | Small Impact | Moderate Impact | Large Impact |
| --- | --- | --- | --- |

|  | 0 | 10 | 20 | 30 | 40 | 50 | 60 | 70 | 80 | 90 | 100 |
| --- | --- | --- | --- | --- | --- | --- | --- | --- | --- | --- | --- |

| What will be the impact of this event on this person's opinion of gluten-free diets? () |  |
| --- | --- |

store11

|  | Small Impact | Moderate Impact | Large Impact |
| --- | --- | --- | --- |

|  | 0 | 10 | 20 | 30 | 40 | 50 | 60 | 70 | 80 | 90 | 100 |
| --- | --- | --- | --- | --- | --- | --- | --- | --- | --- | --- | --- |

| What will be the impact of this event on this person's opinion of gluten-free diets? () |  |
| --- | --- |

End of Block: wstore
