# Supplementary material for: Perceived impact of information signals on opinions about gluten-free diets
Source: PLoS One. 2021 Apr 8;16(4):e0248570. doi: 10.1371/journal.pone.0248570 (PMC8031409; doi:10.1371/journal.pone.0248570)
Supplement: Attachment — Submitted filename: response to all reviewers.docx [file pone.0248570.s002.docx]

**Response to all reviewers**

**Response to Reviewer 1**

*Dear Reviewer:*

*Thank you for your kind and helpful review. We were happy to rewrite the article to address your concerns, making it longer and adding more sophisticated data analysis methods. Allow us to explain why our initial manuscript was so short. It is our belief that scientists should publish more articles that are shorter and easier to read. This particular dataset provided a unique opportunity to do so, as the survey was designed and administered such that the results could be displayed in a simple chart that anyone, regardless of their background, would be able to read. Journals rarely publish short, simple manuscripts, but it is unclear if that is because journals do not want such papers or that they rarely receive them as submissions, so we decided to submit it as a short, simple article to give the editors and reviewers the opportunity to publish such an article if they so desire. This turned out not to be the case, and that is fine, as we are happy to revise the article to make it longer and more sophisticated. We hope you find the revised article improved and worthy of publishing in PLOS ONE.*

*A summary of the changes in the revised manuscript are:*

- *Literature suggested by a reviewer is incorporated into the introduction.*
- *The data analysis now includes*
  - *A simple summary of the average impact scores, like before*
  - *A weighted average of the impact scores, using weights from a sample balancing algorithm to help the sample better represent the nation at-large.*
  - *A regression analysis explaining impact scores as a function of demographics and the hypothetical other person used.*
  - *A similar regression analysis using weights from a sample balancing algorithm.*
  - *Three sensitivity analyses to evaluate how the inclusion of people unfamiliar with gluten impacted the results and how results would change if we allowed for heterogeneity in the regression parameters using a latent class model.*
- *The paper now includes a limitations sections and an extensive discussion section.*

*Below are your comments and suggestions with our responses. At every instance it was possible to comply with your suggestions, we attempted to do so, and we hope we interpreted your questions and suggestions correctly. It is our impression that we come from different fields of social science, so if we misinterpreted something please accept our apology.*

Reviewer #1: The topic tackled by this paper is an interesting one. The influences that have driven the trend around gluten-free diets (outside of diagnosed celiac disease) merits scrutiny. Though I think the value of this study is really in the potential application of this approach to the communication of food related information that has the potential to positively impact people’s diets. I do however have some major concerns about the methodology and the approach to data analysis. I think for this to be publishable standard these need to be revised and I would suggest a new data analysis strategy that better reflects the design of the study and the breadth of the dataset. Finally, I think it would be worth splitting the results and discussion sections and producing a more comprehensive discussion section.
 *As mentioned above, the data are now analyzed using numerous statistical techniques (although the overall results do not not alter). As you requested, there is now an independent and extensive discussion section.*

1. The title feels a bit misleading at the moment. It needs to be made clearer that actual knowledge of attractive people/ doctors was not assessed – it was actually people’s perception of how impactful others would find their knowledge.

*Nice idea. We thus changed the title to “Perceived impact of information signals on opinions about gluten-free diets.”*

2. The introduction would benefit from a strong set of justified hypotheses in addition to the overarching aim stated.

*We comply with this request in a slightly different way, and we hope you approve. We started to comply by listing some hypotheses to be tested, but doing so felt inauthentic and a bit misleading because this is an exploratory analysis, not a confirmatory analysis. Thus, we now explicitly say in the introduction that it is an exploratory analysis, and explicitly remark that we do not begin with a set of hypotheses to be tested.*

3. Materials and methods -should be in past tense not present tense.

*This section is now written in the past tense.*

4. Please provide the exact number of respondents and the exact number of useable responses at line 73 rather than an ‘over 1500’ which is fine for a summary but doesn’t make sense in the context of a methods section.

*We now state we use 1,317 respondents exactly. The number is now lower than 1,500 because now that regression analysis is included involving demographics we excluded any subjects that did not complete all the demographic questions.*

5. Please provide the rationale for the sample size. Was a power analysis conducted?

*We generally never do a power analysis whenever we can survey more than 1,000 people. The reason is that if you use sample size calculators, making reasonable assumptions like a population of 10,000, a confidence level of 95% and a margin of error of 5% you find the optimal sample size to be less than 500 observations. So by using large sample sizes like ours of 1,317 we don’t feel like we need to explicitly defend the sample size, but if we used less than 1,000 respondents we certainly would. In our field, when administering internet surveys which are not terribly expensive, we usually aim for more than 1,000 observations because that is generally thought to be more than enough observations, especially for analyses like this one.*

6. Where did these questions that are referred to throughout the methods section come from? Were they made up specifically for this and how was this done? Was any kind of piloting or validation work engaged in?

*This is an exploratory analysis, so these are questions that we came up with, using signals that we thought were reasonable. If we were attempting to construct a psychometric scale or something similar to that then we would perform some sort of in-sample validation, but this is not really possible in this type of exploratory work. There was substantial piloting of course, where we have have lots of different people take the survey and provide us feedback, but that is always done in a survey so we saw no need to document it.*

7. Line 81: Did you check that they put people in a frame of mind thinking of gluten? If so, how? I would remove that claim otherwise.

*We did remove that claim, thank you.*

8. What did you do with the 4% who had never heard of gluten free foods? Eliminate or retain and why?

*We retained them, but in the revised manuscript we perform a sensitivity analysis to show that the results are the same if you eliminate or retain them. We also performed a number of additional sensitivity analyses.*

9. How were the ten signals developed?

*Based on reason. We had extensive discussions with a number of people about what type of signals would be interesting to explore. It is an exploratory analysis, so we were willing to evaluate signals that we knew would matter (e.g., doctor recommendation) and signals we doubted would matter but had potential (e.g., learning about the official Gluten-Free Day).*

10. Was the scale (small to high impact) piloted or validated in any way?

*No, there was no in-sample validation of the ‘scale’ because we are not constructing a psychometric scale of any kind. We might be thinking of some different types of statistical methods, and we might be coming from different fields with different traditions. The motivation for the use of indirect questioning stems from research in psychology, marketing, and economics. Also, we did not want to ‘validate’ or help define what would be considered a ‘large’ or ‘small’ impact, as we wanted the subject to define this for themselves. Now, it might have been useful to provide additional questions that had a well-defined meaning of a ‘large’ impact, but we did not want to induce respondent fatigue, and this is now mentioned in the new Limitations section.*

11. Where did the pictures come from? How were they selected?

*The pictures were acquired from website that provide royalty free pictures and they were selected to provide a diversity of demographic profiles. We now explain this more in the revised manuscript.*

12. It would be really helpful to divide the methods section into ‘participants, materials, procedure and data analysis’ sections.

*Gladly, the revised manuscript follows your recommendation.*

13. 93-105- this would sit better in the intro section – the overall justification of approach would give more of a justified rationale in the intro section and then the methods section could be more procedural. The nuts and bolts of what happened get a bit lost in the methods section at the moment.

*Now that the paper is considerably longer it follows a more conventional outline, which we think complies with your request above.*

14. There is currently no mention of ethics in the manuscript itself that I could see.

*This study was approved by our university’s Institutional Review Board. This information, along with the IRB application number, is now indicated in the revised manuscript.*

15. I would strongly recommend the results and discussion section be split into two separate sections. At the moment the combined section is quite confusing to follow and it also feels like the two functions of the results and the discussion are not currently adequately met.

*As you requested, these are now two sections. We also provide an extensive discussion of how to think about the results in terms of philosophical theories of truth.*

16. If there were 11 person pictures and 10 signal statements, does that mean that across the sample not everyone saw every person? Doesn’t this mess up the counterbalancing and mean that you cannot really collapse across person for your analyses?

*No, there does not need to be ‘balance’ between persons and signals. We could have conceivably used five person pictures or 50 person pictures. You are right that not everyone saw all persons, but there is no need for every respondent to see every person. Some, due to the random draw of the pictures, may have only seen 7 people. Conceptually, a perfect design would be one where each signal would be matched with the picture of a randomly chosen American from the American population, where the number of persons would be in the hundreds of millions.*

17. Overall, the approach to analysing the data is really disappointing. I really think a more sophisticated approach should be taken and I don’t really agree with this opinion that subjectivity around the scale is a reason to NOT engage in more sophisticated (probably parametric) analyses. It is a well anchored scale and it is not a between subjects design so differences in interpretation should average out? Notwithstanding this, subjectivity around scales is something that could be discussed in a limitations section of a discussion section.

*As mentioned above, we wanted to offer PLOS ONE the option of publishing a short version of the paper if they liked, as we feel there should be more easy to read papers in science and this was a good study to describe succinctly. However, we gladly expand the paper and include more sophisticated anlaysis. In addition to the simple averages used in the first manuscript, we now use sample balancing to obtain weighted averages that better reflect the US population. Also, we estimate two regressions, one using demograpics as explanatory variablesd and one using demographics for sample balancing weights. Moreover, the regressions are reestimated in three different ways to provide a sensivity analysis, one of these being a latent class analysis.*

18. Connected to the last point, it really seems odd not to analyse with ‘person’ as a factor in the analysis. I would strongly recommend that the author looks into more sophisticated ways of analysing data to better reflect the design of the study. Also, it seems a shame not to have taken account of other information collected such as demographics and attitudes of gluten free diets -are their individual differences for example? Can some specific hypotheses be developed in advance of scrutinising the data in this respect?

*The revised manuscript now includes ‘persons’ as an explanatory variable in a regression analysis. This was initially avoided because we deliberately created a survey design that would allow us to exhibit the results in a simple manner without having to perform econometric analysis. Because the persons were randomized in the experiment design, simple averages can be provided without being ‘controlled for’ with regression. However, we gladly include them now.*

*We do include demographic variables as explanatory variables, but with your permission, prefer not to include attitude or knowledge variables regarding gluten because they are likely endogenous to the values of the dependent variables and thus not very informative to study.*

19. Please produce a more straightforward figure depicting key results. The background and extra bits make it difficult to scrutinise from a scientific perspective – attractive though it may look!

*Gladly, the revised figure is less busy.*

20. It doesn’t look like there was any engagement with open science- I would add this to a limitations section in the discussion section.

*We are only vaguely familiar with the open science movement, and from my reading it sounds like a process used in psychology that we do not use in my field. If we listed it as a limitation we believe it would sound more like an advertisement for the open science movement than an actual limitation, so we’d rather not do this, but if you insist we gladly will.*

21. Overall, a discussion section which takes a more critical and less descriptive approach would be welcome. Also, more sophisticated analyses would allow for a deeper consideration of the overarching scientific questions.

*Good idea! For the discussion section we decided to discuss the results from the perspective of philosophy theories of truth to help the reader understand exactly what we can learn from the survey.*

22. Overall, a considered limitations section would be welcome.

*A new section describing limitations of the study is now provided.*

23. Discussion of how this approach could be used on high priority questions around the communication of dietary information might be engaged in.

*This is an interesting idea, and one that we considered exploring. We even drafted some paragraphs doing so. However, no matter how many drafts we made the section always seemed like it would be controversial with reviewers. The only way we were able to be comprehensive enough to please all possible reviewers was to make the section so long that it became basically its own paper. So in short, we tried, but we just couldn’t find a way to do it that we felt would be seen favorably by reviewers. We hope you understand.*

**Response to Reviewer 2**

Dear Reviewer:

Thank you for your kind and helpful review. We were happy to rewrite the article to address your concerns, making it longer and adding more sophisticated data analysis methods. Allow us to explain why our initial manuscript was so short. It is our belief that scientists should publish more articles that are shorter and easier to read. This particular dataset provided a unique opportunity to do so, as the survey was designed and administered such that the results could be displayed in a simple chart that anyone, regardless of their background, would be able to read. Journals rarely publish short, simple manuscripts, but it is unclear if that is because journals do not want such papers or that they rarely receive them as submissions, so we decided to submit it as a short, simple article to give the editors and reviewers the opportunity to publish such an article if they so desire. This turned out not to be the case, and that is fine, as we are happy to revise the article to make it longer and more sophisticated. We hope you find the revised article improved and worthy of publishing in PLOS ONE.

A summary of the changes in the revised manuscript are:

- Literature suggested by a reviewer is incorporated into the introduction.
- The data analysis now includes
  - A simple summary of the average impact scores, like before
  - A weighted average of the impact scores, using weights from a sample balancing algorithm to help the sample better represent the nation at-large.
  - A regression analysis explaining impact scores as a function of demographics and the hypothetical other person used.
  - A similar regression analysis using weights from a sample balancing algorithm.
  - Three sensitivity analyses to evaluate how the inclusion of people unfamiliar with gluten impacted the results and how results would change if we allowed for heterogeneity in the regression parameters using a latent class model.
- The paper now includes a limitations sections and an extensive discussion section.

Below are your comments and suggestions with our responses. At every instance it was possible to comply with your suggestions, we attempted to do so, and we hope we interpreted your questions and suggestions correctly. It is our impression that we come from different fields of social science, so if we misinterpreted something please accept our apology.

Reviewer #2: 1) Is the manuscript technically sound, and does the data support the conclusions?

In places yes, in other areas it seems very brief and could be improved.

*As mentioned above, the manuscript is much longer and detailed now.*

There is no detail of survey method and indirect questioning in abstract.

*Very good point! This is information that should be in the abstract but was not. The revised manuscript now include the information in the abstract, though. Thank you.*

The introduction gives a very simplistic overview of non coeliac gluten sensitivity and the controversy around this area - this lacks depth in my opinion and needs to be improved.

There's no mention of the criteria outlined for diagnosing NCGS see

Catassi et al. (2015) . Nutrients 7:4966-4977.


or of RCT that have found an effect from gluten - e.g.


Shahbazkhani et al (2015) Non-Celiac Gluten Sensitivity Has Narrowed the Spectrum of Irritable Bowel Syndrome: A Double-Blind Randomized Placebo-Controlled Trial. Nutrients (7) 4542-4554.

*We gladly include these two references and an expanded discussion. We initially did not include them because we thought MDPI was a predatory journal, but we now see they are no longer considered one so we are happy to include them. Note that our study is not about actual diagnoses of gluten sensitivity, but regular people’s beliefs that gluten is in general ‘bad’.*

I am unclear if the survey had ethical approval and if participants gave consent

*Yes, our university’s Institutional Review Board did approve this research and this information is now included in the revised manuscript.*

Or that Challenge amount used in gluten challenge is likely to be key re findings gluten challenge - This paper reports it's fructans rather than gluten but they use an amount for gluten challenge that is below what's recommended in the Catassi et al 2015 paper - so it is that surprising that they find no effect?

- Skodje GI, Sarna VK, Minelle IH, et al. Fructan, Rather Than Gluten, Induces Symptoms in Patients With Self-Reported Non-Celiac Gluten Sensitivity. Gastroenterology. 2018;154(3):529-539.e2. doi:10.1053/j.gastro.2017.10.040

*Thank you! We overlooked this very relevant article.*

Writing around weight loss @ line 49 is overly simplistic too. Excluded 'free from' diets are perceived by many to aid weight loss as they may restrict many foods that are treats and high in calories (ie. biscuits, cakes, sweets). Therefore there is evidence they are perceived as useful for weight loss.

*Now that the paper is longer we actually discuss this in the new Discussion section and shorten the sentence you mentioned.*

You may find this paper a useful reference to motivations for following a gluten free diet in absence of coeliac disease.


Harper, L., Bold, J. (2018). An exploration into the motivation for gluten avoidance in the absence of coeliac disease. Gastroenterology and Hepatology from Bed to Bench 11 (3): 259-268.

*We now cite this article, thank you.*


More depth around responses would be useful too. E.g. Line 76-78 were responses representative geographically? Was this considered ? E.g. Rural v city areas, E / W coast versus central states?

*As you will see there is now a very intense data analysis, which includes a geographic analysis.*
